# Parliament and Publications

**Published:** 1910-12-03

**Authors:** 


### Parliament and Publications.

### SLEEPING SICKNESS IN NYASALAND.

In reply to a question by Sir J. D. Rees, the Secretary of State for the Colonies stated that he had received information of nine cases of sleeping sickness in Nyasaland, and there seemed to be strong evidence that *Glossina palpalis* does not exist in the area in which they occurred.

### A TYPHOID FEVER CARRIER.

It has now been discovered that the typhoid fever which has been more or less prevalent in the Folkestone urban district for many years has been spread mainly by milk infected by a typhoid carrier. He worked at five farms between April 1893 and October 1909; during his employment cases of fever occurred at three of the farms, and more than half the cases between 1896 and 1909 occurred in households in which milk from the farm at which the "carrier" was at the time working was being consumed. The case is dealt with in a report by Dr. R. W. Johnstone, issued by the Local Government Board.

### OSBORNE.

The report by the House Governor and medical superintendent for the year ending March 31, 1910, shows that the total number of officers admitted during that period was two hundred and of ladies sixty-nine. This is a decrease on the previous year, which is accounted for by (1) insufficient knowledge of the benefits of Osborne among sick officers; (2) the very large reduction in the invaliding home of officers; (3) the establishment of nursing homes and institutions for sick officers in the hills in India, where many cases pass their period of convalescence instead of coming home. The largest number of admissions during the year were for dysentery, neurasthenia, and diseases of the digestive system. The cases of neurasthenia nearly all required more or less complete courses of Weir Mitchell treatment. The general results in these cases were excellent, considering their nature, and the usually prolonged duration of the previous illness prior to treatment.—"Osborne," Cd. 5414, price 6d.

